# Altered brain activity in end‐stage knee osteoarthritis revealed by resting‐state functional magnetic resonance imaging

**DOI:** 10.1002/brb3.2479

**Published:** 2021-12-29

**Authors:** Bing‐Xin Kang, Jie Ma, Jun Shen, Hui Xu, Hai‐Qi Wang, Chi Zhao, Jun Xie, Sheng Zhong, Chen‐Xin Gao, Xi‐Rui Xu, Xin‐Yu A, Xiao‐Li Gu, Lianbo Xiao, Jianguang Xu

**Affiliations:** ^1^ The First Affiliated Hospital of Henan University of Chinese Medicine Zhengzhou China; ^2^ School of Rehabilitation Science Shanghai University of Traditional Chinese Medicine Shanghai China; ^3^ Guanghua Hospital Shanghai University of Traditional Chinese Medicine Shanghai China; ^4^ Arthritis Institute of Integrated Traditional Chinese and Western Medicine Shanghai Academy of Traditional Chinese Medicine Shanghai University of Traditional Chinese Medicine, Shanghai, China; ^5^ Henan University of Chinese Medicine Zhengzhou China

**Keywords:** brain activity, functional connectivity, knee osteoarthritis, resting‐state functional magnetic resonance imaging, voxel‐based morphometry

## Abstract

**Introduction:**

Knee osteoarthritis (KOA) is characterized by a degenerative change of knee cartilage and secondary bone hyperplasia, resulting in pain, stiffness, and abnormal walking gait. Long‐term chronic pain causes considerable cortical plasticity alternations in patients. However, the brain structural and functional alterations associated with the pathological changes in knee joints of end‐stage KOA patients remain unclear. This study aimed to analyze the structural and functional connectivity alterations in end‐stage KOA to comprehensively understand the main brain‐associated mechanisms underlying its development and progression.

**Methods:**

In this study, 37 patients with KOA and 37 demographically matched healthy controls (HCs) were enrolled. Alternations in gray matter (GM) volume in patients with KOA were determined using voxel‐based morphometry. The region with the largest GM volume alteration was selected as the region of interest to calculate the voxel‐wise resting‐state functional connectivity (rs‐FC) in the two groups. Pearson's correlation coefficient was used to analyze the correlation between clinical measures and GM volume alternations in patients with KOA.

**Results:**

Compared with HCs, patients with KOAs exhibited significantly decreased GM volumes in the left middle temporal gyrus (left‐MTG) and the left inferior temporal gyrus. Results of the voxel‐wise rs‐FC analysis revealed that compared with HCs, patients with KOA had decreased left‐MTG rs‐FC to the right dorsolateral superior frontal gyrus, left middle frontal gyrus, and left medial superior frontal gyrus. GM volume in the left‐MTG was negatively correlated with the Western Ontario and McMaster Universities Arthritis Index in patients with KOA (*r* = −0.393, *p* = .016).

**Conclusion:**

Structural remodeling and functional connectivity alterations may be one of the central brain mechanisms associated with end‐stage KOA.

## Graphical abstract

Neuroimaging study in end‐stage knee osteoarthritis.

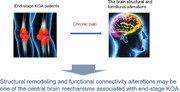

## 1 INTRODUCTION

There is a phenomenon of central sensitization in patients with osteoarthritis (Fingleton et al., 2015; Sofat et al., 2013), as the central nervous system is structurally altered by the process of regulation of nociceptive pain stimuli (Gwilym et al., 2009). Previous studies have reported that chronic pain affects the brain structure and function in patients with back pain (Apkarian et al., 2004; Tagliazucchi et al., 2010), fibromyalgia (Čeko et al., 2020; Kuchinad et al., 2007), multiple sclerosis (Bosma et al., 2018), and carpal tunnel syndrome (Maeda et al., 2016), among others.

End‐stage knee osteoarthritis (KOA) is primarily caused by degenerative changes in the articular cartilage and secondary hyperosteogeny, which cause chronic pain, stiffness, and abnormal walking gait (Baumbach et al., 2020). These pathological changes influence the spatial patterns of intrinsic brain activity in patients. Ushio et al. found that in female patients with severe KOA, the anterior insular cortex shows stronger rest‐state functional connectivity (rs‐FC) with the right orbitofrontal cortex, subcallosal area, and bilateral frontal pole than in healthy controls (HCs), and the extent of this increase is substantially associated with disease‐specific measurement (Ushio et al., 2020). The functional alteration of chronic pain in KOA involves cerebral cortex remodeling associated with changes in the cortico‐cortical and cortico‐subcortical pathways (Cauda et al., 2014; Hiramatsu et al., 2014). Barroso et al. reported that precentral cortex gray matter (GM) volume is lower in patients with KOA than HCs (Barroso et al., 2020). Liao et al. (2018) reported that patients with KOA displayed markedly decreased regional GM volume in various regions, including the bilateral orbital frontal cortex and right lateral prefrontal, precentral, and postcentral cortices compared with HCs. Lewis et al. (2018) found that KOA decreases GM volume bilaterally in the amygdala, nucleus accumbent, and ipsilateral primary somatosensory cortex compared with HCs.

Patients with KOA experience intense resting pain (Power et al., 2018), which reduces their activity, decreases cognitive function, and increases the risk of developing dementia (Huang et al., 2015). A correlation exists between cortical changes and KOA‐modified motor behavior (Shanahan et al., 2015). Thus, it is significant to elucidate the neurobiological mechanisms underlying the development of end‐stage KOA in order to prevent the occurrence and development of secondary mental diseases.

Resting‐state functional magnetic resonance imaging (rs‐fMRI) is one of the most widely used methods in neuroimaging research as it can generate high‐resolution images. In addition, it is noninvasive, which increases its ease of use in the clinical setting (Zuo & Xing, 2014). The realization of advanced cognitive functions of the brain depends on the collaborative cooperation between different brain areas, not just on an individual brain region. Functional connectivity can reflect indirect links between brain regions (Adachi et al., 2012). Unlike previous studies, we analyzed the structural abnormalities and functional connectivity alterations in end‐stage KOA to obtain a comprehensive understanding of the main brain‐associated mechanisms underlying its development. First, we used voxel‐based morphometry (VBM) to determine the differences in GM volume between patients with end‐stage KOA and HCs. Second, the region exhibiting the largest GM volume alteration was selected as the region of interest (ROI), following which differences in rs‐FC to the ROI and whole brain were observed in patients with KOA and HCs. Finally, the correlation between GM volumes and clinical measurements was statistically assessed in patients with KOA. Compared with the technique of selecting ROIs based on prior hypotheses, the method devised in the present study has the advantage of evaluating changes in brain function based on a hypothesis generated from the same participants but using a complementary imaging method.

## 2 MATERIALS AND METHODS

### 2.1 Subjects

This prospective case–control study was approved by the Medical Ethics Committee of Guanghua Hospital, Shanghai University of Chinese Medicine, and was registered in the Chinese Clinical Trial Registry (ChiCTR2000033778). The study was conducted from December 2020 to May 2021 at Guanghua Hospital, Shanghai University of Traditional Chinese Medicine, Shanghai, China.

Inclusion criteria for end‐stage KOA were as follows: (i) patients with KOA classified as being in stage III or IV via Kellgren–Lawrence classification (Kohn et al., 2016); (ii) patients with pain that could not be effectively relieved after nonsurgical treatment; and (iii) patients who did not undergo invasive treatment in the past 3 months. Exclusion criteria were as follows: (i) patients who had undergone other surgical procedures; (ii) patients who were afflicted by neurological or psychiatric disorders; (iii) patients who had a history of head trauma/neurodegenerative illness or seizure disorder; (iv) patients who had contraindications to rs‐fMRI; or (v) patients who were unable to tolerate rs‐fMRI scanning.

HCs were selected through a yoked review process that matched the age, education, sex, and ethnicity of individual surgery participants. All participants received Montreal Cognitive Assessment (MoCA) system assessments on the day prior to rs‐fMRI scans. The assessment areas included attention and concentration, executive function, memory, language, visual structure skills, abstract thinking, and calculation and orientation skills (30 points in total).

### 2.2 Structural and functional MRI data acquisition

The rs‐fMRI data were acquired using a Clinical 1.5 Tesla whole‐body magnetic resonance imager (United Imaging, Shanghai, China). Head huggers and earplugs were used to minimize noise and limit head movement while performing rs‐fMRI scanner examinations. Subjects were instructed to keep their eyes closed, relax, stay awake, and not think about anything specific. Rs‐fMRI images were generated using a rapid‐gradient echo‐planar imaging sequence with the following settings: repetition time, 3000 ms; echo time, 30 ms; a flip angle, 90°; a field of view, 225 × 225 mm^2^; acquired matrix, 64 × 64 matrices; thickness (of 43 slices), 3.5 mm; voxel size, 3.52 × 3.52 × 3.52 mm^3^; and bandwidth, 2250 Hz/pixel. The scan lasted 12 min and 13 s. In their response to a simple questionnaire that was filled after the scan, all subjects stated that they had not fallen asleep. Three‐dimensional T1‐weighted magnetization‐prepared rapid‐gradient echo sagittal images were captured using the following parameters: repetition time, 10.4 ms; echo time, 4.4 ms; an inversion time, 750 ms; flip angle, 10°; 256 × 232 matrix resolution; and voxel size, 1 × 1 × 1 mm^3^. The scan lasted 3 min and 32 s.

### 2.3 Statistical processing and analysis

#### 2.3.1 Clinical data analysis

Baseline demographic information was analyzed using SPSS V.25.0 (IBM, Armonk, NY, USA). Two‐sample *t*‐tests or χ^2^ tests were performed between two groups as appropriate. Continuous variable data are represented by the mean ± standard deviation (SD). Statistical significance was set at *p *< .05.

#### 2.3.2 Structural image processing for voxel‐based morphometry analysis

Structural image analysis was performed using MATLAB R2013a (Math Works Inc. Natick, MA, USA) with an automated Computational Anatomy Toolbox (CAT 12, http://www.neuro.uni‐jena.de/cat/) based on Statistical Parametric Mapping (SPM 12, http://www.fil.ion.ucl.ac.uk/spm/software/spm12/). The processes consisted of the following steps: (1) Digital Imaging and Communications in Medicine (DICOM) files were completely converted to nii.gz format using MRIConvert (https://lcni.uoregon.edu/downloads/mriconvert). (2) The high‐resolution structural images were segmented into GM, white matter (WM), and cerebrospinal fluid (CSF), bone, nonbrain soft tissue, and background using International Consortium for Brain Mapping Tissue Probabilistic Atlases provided by SPM 12. All images passed a visual quality check for image quality and possible movement artifacts and homogeneity control. (3) The Diffeomorphic Anatomical Registration Through Exponentiated Lie (DARTEL) algebra was used in SPM 12 to perform anatomical and morphological registration, normalization, and modulation analysis (Ashburner, 2007). All GM images were aligned and resampled to a size of 1.5 × 1.5 × 1.5 mm^3^ and then normalized to Montreal Neurological Institute (MNI 152) space. Segmented images of the GM were modulated to reflect volume measurement using the inverse Jacobian matrix of local transformation. (4) The normalized and modulated images were subjected to smoothing using an isotropic Gaussian kernel of 8 mm full width at half maximum.

Statistical analysis of the structural image was performed using age and global GM volume as covariates of no interest. Between‐group GM volume changes were investigated by means of a two‐sample *t*‐test. The statistical significance of differences between groups was set at a cluster‐level AlphaSim corrected *p *< .05 combined with uncorrected voxel‐wise *p *< .001.

#### 2.3.3 Functional image processing

Functional images analysis was performed using the Data Processing Assistant for rs‐fMRI (REST 1.8). This toolbox is based on SPM 12 and runs using MATLAB R2013a. The processing included the following steps: (1) All scans were converted from DICOM to nii format using MRIConvert. (2) The first 10 volumes of images were discarded to allow the signal to each equilibrium and the subjects to adapt to the scanning noise. (3) Slice‐time correction was performed to compensate for the acquisition time delay between each slice. (4) The corresponding structural images of the subjects were corrected, realigned, and co‐registered to correct the motion between time points. Head motion parameters were computed by estimating the translation in each direction and the angular rotation on each axis for each volume. All functional images acquired from all participants were within the defined motion thresholds (translational or rotational motion parameters less than 3 mm or 3°). (5) In the normalization step, individual structural images were first co‐registered with the mean functional image; next, the transformed structural images were segmented and normalized to the MNI space using a high‐level nonlinear warping algorithm (the DARTEL technique) (Ashburner, 2007). (6) To improve the signal‐to‐noise ratio and ensure that the data satisfy the properties of random Gaussian field, spatial smoothing was performed using a Gaussian kernel of 6‐mm full width at half maximum. (7) Linear trend subtraction was performed to remove noise from instability during the inspection and to determine baseline drift and signal float. (8) Regression covariates were also obtained. This step is performed to regressor in the signals of WM and CSF in the brain to reduce their interference with the GM signal. (9) Temporal filtering (0.01−0.08 Hz) was performed on the time series of each voxel for rs‐FC analysis. This step removes physiological noises, including breathing, heartbeat, and involuntary movement.

The mask of ROI was extracted from the automated anatomical labeling atlas. Contrast images were generated for each subject by estimating the regression coefficient among all brain voxels. We first localized the significant area in rs‐FC maps of each group using a one‐sample *t*‐test. Next, we prepared a binary mask, as applicable. We used the two‐sample *t*‐test to determine the difference in voxel‐wise rs‐FC between patients with KOA and HCs (control covariates: age). The correlation coefficient map was then converted into a Fisher‐z map using Fisher's r‐to‐z transformation to improve normality. The statistical significance of differences was set at a cluster‐level AlphaSim corrected *p *< .05 combined with uncorrected voxel‐wise *p *< .001.

#### 2.3.4 Correlation between GM volume and Western Ontario and McMaster Universities Arthritis Index

The Western Ontario and McMaster Universities Arthritis Index (WOMAC) is widely used for evaluating pain, stiffness, and physical function in patients with KOA (Bellamy et al., 2011). The correlation between GM volume and WOMAC scores was determined using an ROI analysis, and the REST toolbox was used to extract the value of GM volume in patients with KOA. Pearson correlation analysis was used to explore the relationship between WOMAC scores and GM volume using SPSS V.25.0.

## 3 RESULTS

### 3.1 Demographics and clinical data

Demographic data of the subjects are presented in Table 1. There were no significant differences between patients with KOA and HCs in terms of age, gender, and MoCA scores.

**TABLE 1:** Demographic characteristics and clinical data between KOA patients and HCs

**Characteristic**	**End‐stage KOA**	**Healthy controls**	***p*‐value**
VBM (*n*)	37	37	/
Male/female	3/34	7/30	.177^a^
Age (mean ± SD, years)	71.6 ± 5.6	69.5 ± 5.1	.098
MoCA scores	28.3 ± 1.3	28 ± 1.3	.527
WOMAC	130.0 ± 9.2	—	—
FC (*n*)	23	29	/
Male/female	3/20	7/22	.318^a^
Age (mean ± SD, years)	73.2 ± 5.5	69.4 ± 5.3	.958
Total intracranial volume (mean ± SD, mL)	1305.7 ± 120.6	1280.8 ± 88.6	.314

*Abbreviations*: FC, functional connectivity; HCs, healthy controls; KOA, knee osteoarthritis; MoCA, Montreal Cognitive Assessment; VBM, voxel‐based morphometry; WOMAC, The Western Ontario and McMaster Universities Osteoarthritis Index.

^a^ Mann–Whitney U test.

### 3.2 Difference between the GM volumes in patients with KOA and HCs

**FIGURE 1:**
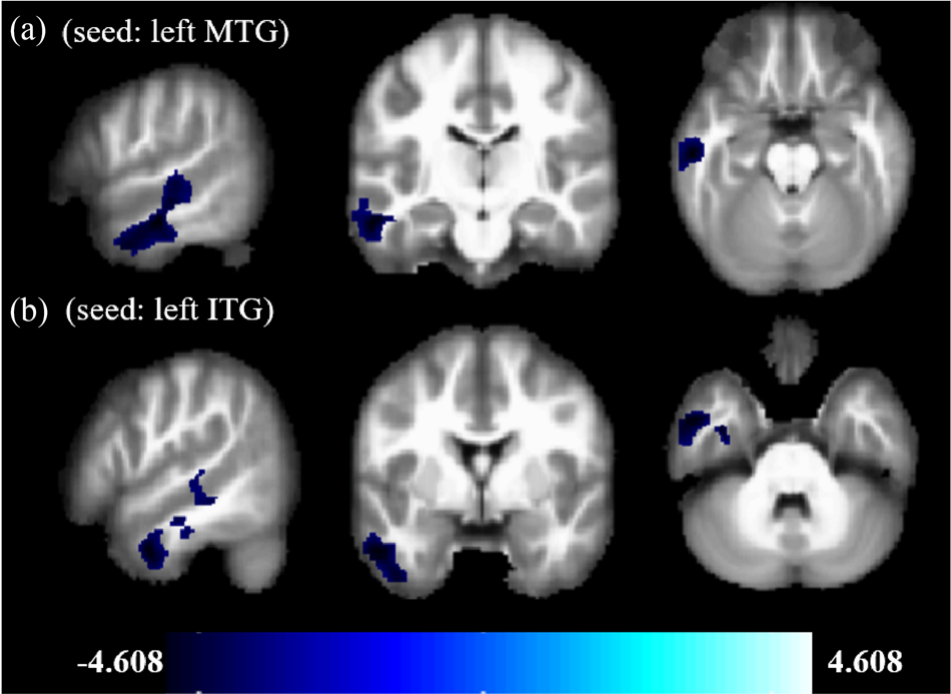
The differences between the gray matter volume of KOA patients and HCs based on voxel‐based morphometry analysis. Brain regions with significantly decreased gray matter as indicated by the *t*‐statistic (blue). Corrections for multiple comparisons were applied (cluster‐level extent = 255 voxels, *p *< .001, AlphaSim correction, *p* < .05, two tailed). KOA, knee osteoarthritis; HCs, healthy controls

Compared with HCs, GM volume decreased in the left middle temporal gyrus (left‐MTG; −57, −18, −21, *T* = 4.610 and −63, −21, −3, *T* = −4.298) and left inferior temporal gyrus (left‐ITG; −54, 0, −36, *T* = 4.510) in patients with KOA. The left‐MTG and left‐ITG belonged to the same cluster (Figure 1 and Table 2). No other regions exhibited an increase in GM volume.

**TABLE 2:** GM volume differences between KOA subjects and HCs

			**Peak MNI**			
**Brain regions**	**Side**	**Volumes (mm^3^)**	***X***	***Y***	***Z***	***t*‐value**
Middle temporal gyrus	L	290	−57	−18	−21	− 4.610
			−63	−27	−3	− 4.298
Inferior temporal gyrus	L		−54	0	−36	−4.510

Coordinates (*X*, *Y*, *Z*) refer to the peak MNI coordinates of brain regions with peak intensity. The resulting statistical map was set at *p* < .05 (AlphaSim correction for multiple comparisons, with combined individual voxel *p* < .001 with a cluster size > 255 voxels).

*Abbreviations*: GM, grey matter; HCs, healthy controls; KOA, knee osteoarthritis; MNI, Montreal Neurological Institute.

### 3.3 The rs‐FC to left‐MTG

**FIGURE 2:**
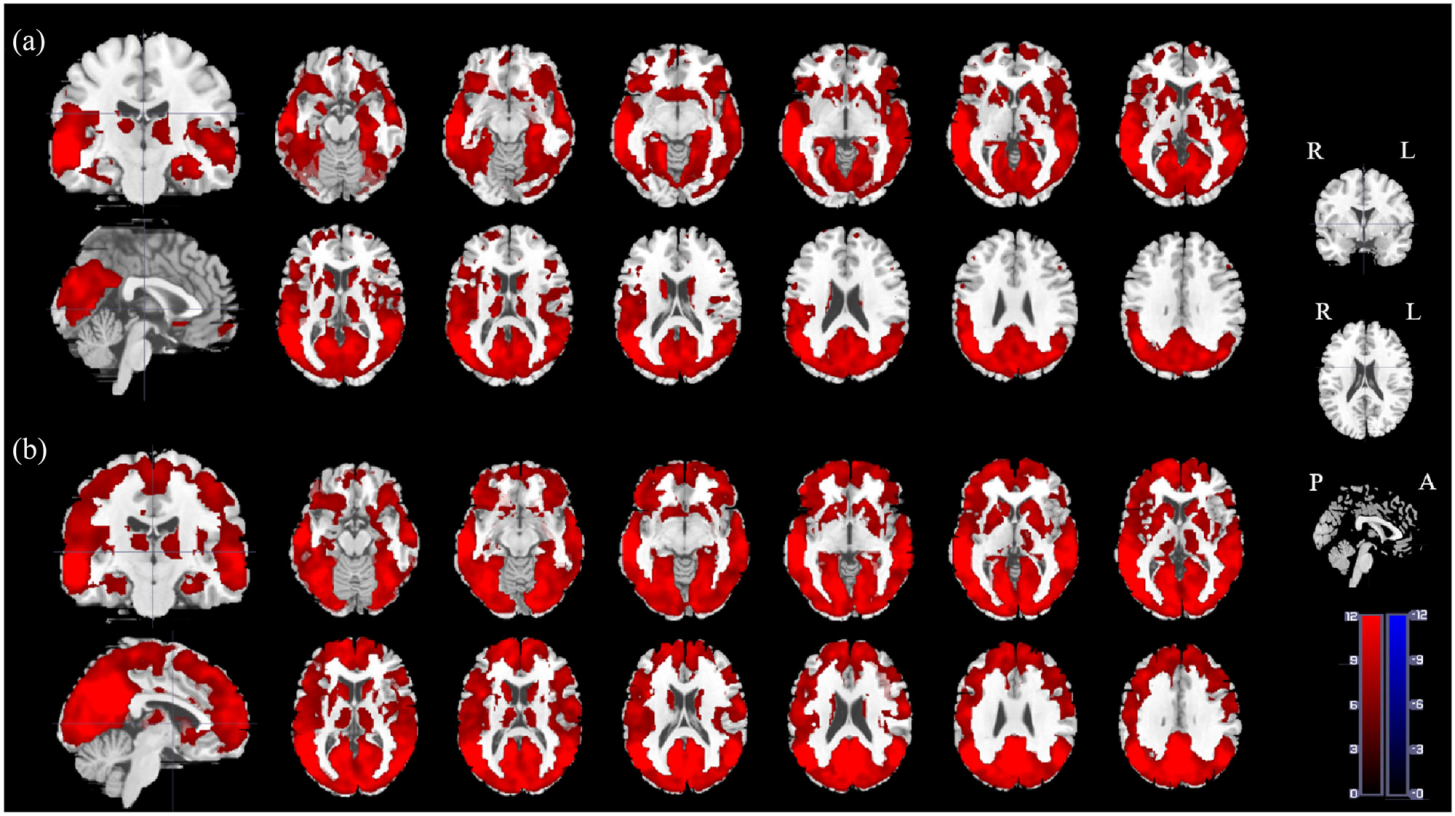
Red represents the area that shows positive connectivity with the left‐MTG.(a) One‐sample *t‐*test results on connectivity between the left‐MTG and other brain regions in KOA patients. (b) One‐sample *t*‐test results on connectivity between the left‐MTG and other brain regions in HCs. KOA, knee osteoarthritis; HCs, healthy controls

**FIGURE 3:**
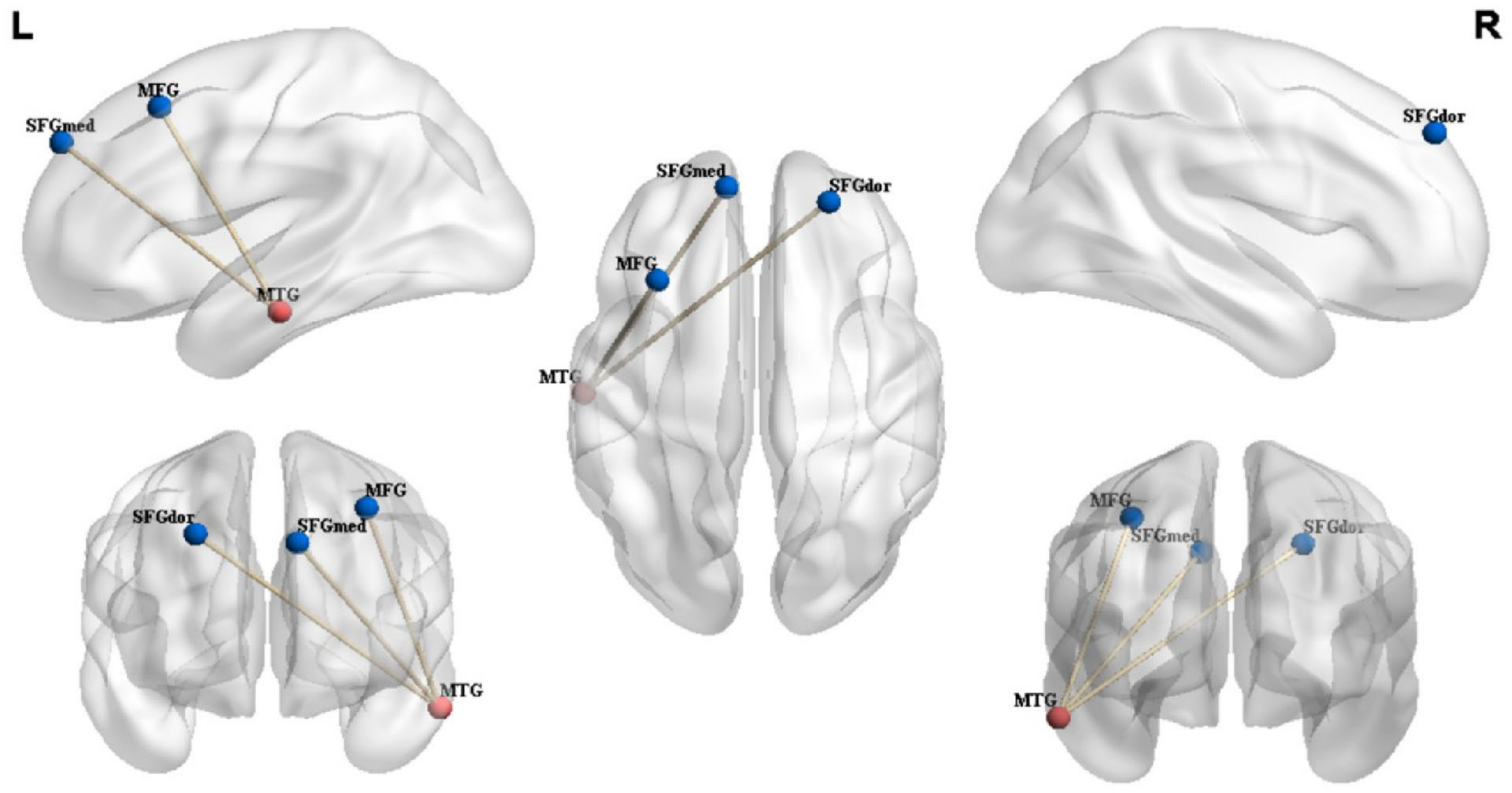
Differences between functional connectivity of KOA patients and HCs. The seed was extracted via the automated anatomical labeling atlas. MTG, middle temple gyrus; SFGdor, dorsolateral superior frontal gyrus; MFG, middle frontal gyrus; SFGmed, medial superior frontal gyrus; L, left; R, right; KOA, knee osteoarthritis; HCs, healthy controls

Within‐group analysis revealed that, in the HC group, the rs‐FC to several regions related to pain/emotions, including the left precuneus, right inferior temporal gyrus, right insula, and left support motor area, were significantly enhanced (Figure 2a). In the KOA group, rs‐FC patterns of the left‐MTG had relatively smaller cluster sizes and included the left angular gyrus, right superior temporal gyrus, and right superior frontal gyrus (Figure 2b). In the between‐group analysis, compared to HCs, the KOA group exhibited decreased rs‐FC in the left‐MTG to the superior frontal gyrus (right‐SFG), left middle frontal gyrus (left‐MFG), and left medial superior frontal gyrus (left‐SFGmed). The three brain regions were part of one cluster (Table 3 and Figure 3). No significantly increased left‐MTG rs‐FC were found in other brain regions.

**TABLE 3:** Regions exhibiting left‐MTG functional connectivity analysis between KOA patients and HCs

			**Peak MNI**			
**Brain regions**	**Side**	**Cluster size**	***X***	***Y***	***Z***	***t*‐value**
Superior frontal gyrus	R	1135	24	48	39	−5.891
Middle frontal gyrus	L		−33	21	48	−5.495
Medial superior frontal gyrus	L		−6	57	36	−5.381

Coordinates (*X*, *Y*, *Z*) refer to the peak MNI coordinates of brain regions with peak intensity. The resulting statistical map was set at *p* < .05 (AlphaSim correction for multiple comparisons, with combined individual voxel *p* < .001 with a cluster size > 392 voxels).

*Abbreviations*: L, left; MTG, middle temporal gyrus; R, right.

### 3.4 Correlations

**FIGURE 4:**
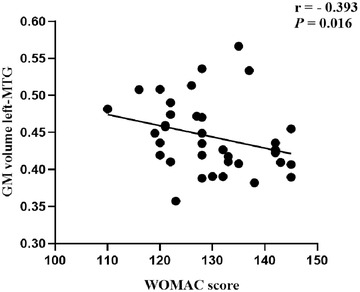
Correlation between gray matter volume in the left‐MTG and WOMAC scores. left‐MTG, left middle temporal gyrus; WOMAC, the Western Ontario and McMaster Universities Osteoarthritis Index

Correlation analysis revealed that the left‐MTG values were negatively correlated with the WOMAC scores (*r* = −0.393; *p* = .016; 95% CI = −0.6421 to 0.06954) in KOA group (Figure 4).

## 4 DISCUSSION

We investigated the alternations in regional brain morphology, as well as rs‐FC in end‐stage KOA patients. VBM analysis, which utilizes structural MRI scans, has been widely and successfully used to identify regional differences in relative GM volumes (Ashburner & Friston, 2001). VBM is recognized as an important tool to detect in vivo morphological changes at high resolution (Höflich et al., 2017). Based on VBM analysis, we found that GM volumes in the left‐MTG and left‐ITG of Chinese patients with end‐stage KOA were significantly decreased compared with those of HCs. However, the exact cause of the volume change in GM is not known currently. Morphological differences under chronic pain conditions are usually associated with the duration and severity of pain experienced by individuals (Barroso et al., 2020; Lewis et al., 2018; Weerasekera et al., 2021). Our results are different from those obtained in previous studies, and this may be due to the differences in the stage of disease and heterogeneity in pain and treatment methods used between the studies. This disagreement in results can also be attributed to differences between the imaging parameters of VBM and sensitivity of scanning machines, as well as the limitations of analyzing details, including the spatially normalizing atypical and robustness of standard parametric tests (Mechelli et al., 2005).

The MTG is the ‘‘core’’ structure of the default mode network (DMN) (Buckner et al., 2008) and this was confirmed in our results (Figure 1). The temporal lobe is known to be related to attention orientation (Hu et al., 2020; Wang et al., 2008) and is a major contributor to pain perception in Fibromyalgia (Schreiber et al., 2017). Inferior temporal gyrus functions are associated with memory, social cognition, action observation, and multimodal sensory integration. Krose et al. (2011) reported that greater self‐reported anxiety is inversely related to the inferior temporal cortex volume in posttraumatic stress disorder. Although the MTG and inferior temporal gyrus are not directly involved in the regulation of pain, changes in GM volume may be related to the comorbidities of chronic pain, such as cognitive and emotional impairment (Smallwood et al., 2013). Proprioception, the conscious or unconscious perception of the position and movement of limbs or joints in space (Knoop et al., 2011), is vital for the regulation of complex and precise joint movements and plays a key role in KOA (Knoop et al., 2011). The WOMAC is a scale used to assess the functional status of the knee joint in patients. Higher the score, the worse the knee function is in patients with KOA. We found that the degree of atrophy of the left‐MTG of patients with KOA shows a negative correlation with the functional status of the knee joint, which could be a direct result of severe knee dysfunction accompanied by an abnormality in pain perception. Altered functional connectivity in DMN regions may be an indication of the dominance of cognitive self‐monitoring and the effect of pain in some patients with chronic pain (Buckner & DiNicola, 2019).

Various types of clinical chronic pain conditions are associated with rs‐FC changes within the DMN (Baliki et al., 2014). The DMN is a pathological target for all chronic pain (Cauda et al., 2014). A direct linkage between DMN/insula connectivity and pain intensity is more evident when patients also suffer from highly negative affect. In chronic low back pain patients, increased information transfer between anterior insula and DMN, and its consequent association with clinical pain, is strongly influenced by pain catastrophizing (Kim et al., 2019). Chronic pain has a widespread impact on brain function and changes the temporal and spatial characteristics of DMN, thus altering the dynamics of the brain regions unrelated to pain (Alshelh et al., 2018; Baliki et al., 2008; Tagliazucchi et al., 2010). In our study, we found that the patients exhibited decreased rs‐FC in the left‐MTG to the right‐SFG, left‐MFG, and left‐SFGmed, and these brain regions are located in the prefrontal cortex. The prefrontal cortex is a neuroanatomical brain area related to executive function, cognition, regulation of pain signals, and reward mechanisms (Alagapan et al., 2019; Li et al., 2013; Marsh et al., 2012; Smolker et al., 2015; ), and mediation of emotion regulation (Wang et al., 2018a). Hiramatsu et al. reported that a significant increase in the activity of the dorsolateral prefrontal cortex in KOA is involved in the suppression of chronic pain (Hiramatsu et al., 2014). Anatomically, the SFGdor and SFGmed are located in the upper part of the prefrontal cortex. The SFGdor and SFGmed are both parts of the executive control network (ECN), and in the DMN, are involved in the perception and regulation of pain/emotions (Buckner & DiNicola, 2019; Grieve et al., 2013; Rathbun et al., 2020; Schreiber et al., 2017; ). In patients with KOA, chronic pain induces abnormal brain connectivity between the dorsal prefrontal lobe and the pain matrix (Hiramatsu et al., 2014). Cottam et al. (2018) suggested that the right anterior insula is the key region that drives static and dynamic network connectivity in KOA patients with chronic pain, wherein reduced clinical pain is associated with greater intrinsic sensorimotor network connectivity. Left‐MFG is also involved in the perception of negative emotions (Wang et al., 2018), attributed to a variety of other cognitive functions. Factors associated with cognitive, emotional, and environmental stressors likely modulate chronic pain (Bushnell et al., 2013; Niddam et al., 2017). Patients with chronically severe KOA usually experience negative emotions including anxiety and depression (Rathbun et al., 2020). Decreased rs‐FC may lead to impaired communication between the motor or sensory information centers in the brain, impaired response and attention, and reduced cognitive ability and motor function in older individuals. This may be one of the brain's central mechanisms in end‐stage KOA. We speculated that these factors together reduced the rs‐FC between left‐MTG and ECN, resulting in pain sensitization in KOA patients. However, further investigation is required to ascertain how these factors affect pain in KOA patients, and the mechanisms underlying the connection between the brain centers and the periphery.

This was an exploratory neuroimaging study with the following limitations. (1) As is typical for any cross‐sectional study, we could not explain the interactions between KOA and cerebral abnormalities; thus, longitudinal studies should be designed to answer this question. (2) Sample size was small. The fact that the ages and genders of the HCs did not perfectly match those of the synkinesis patients limited the interpretability of our potential conclusions. It is recommended that a larger number of subjects be recruited for such studies than those enrolled in this study. (3) We did not include the duration of knee pain in patients in this study, as most patients could not provide an accurate starting time of the knee pain. (4) Although we required that KOA patients avoid any analgesic treatment 2 days before the examination, the patients did use some medication or nonpharmacological analgesics during the long course of the disease, which might have confounded any observed brain morphological and functional changes (Schmidt‐Wilcke et al., 2006). (5) Patients with chronically KOA experience negative emotions (Rathbun et al., 2020), and depression and anxiety were assumed to be present; however, we did not conduct depression and anxiety surveys to assess the mental state of the patients, this was one of the limitations of this study. The absence of psychiatric illness assessment was a severe limitation.

## 5 CONCLUSION

Structural remodeling and functional connectivity alternations may be one of the central brain mechanisms associated with end‐stage KOA patients.

## CONFLICT OF INTEREST

The authors declare that they have no conflict of interest.

## AUTHOR CONTRIBUTIONS

Bing‐Xin Kang, Jie Ma, and Jun Shen conceived the study; Bing‐Xin Kang drafted the study; Hui Xu, Hai‐Qi Wang, Jun Xie, Sheng Zhong, Chen‐Xin Gao and Xin‐Yu A recruited the participants. Xi‐Rui Xu and Xiao‐Li Gu collected clinical data. Chi Zhao was responsible for statistical analyses and tables. Lianbo Xiao and Jianguang Xu have primary responsibility for the final content. All authors contributed to writing and revising the paper and agreed to submission.

### PEER REVIEW

The peer review history for this article is available at https://publons.com/publon/10.1002/brb3.2479

## Data Availability

The datasets used and analyzed during the current study are available from the corresponding author on reasonable request.
